# Current Input Pixel-Level ADC with High SNR and Wide Dynamic Range for a Microbolometer

**DOI:** 10.3390/s21072354

**Published:** 2021-03-28

**Authors:** Jeongho Lee, Ilku Nam, DooHyung Woo

**Affiliations:** 1 School of Information, Communications and Electronics Engineering, The Catholic University of Korea, Gyeonggi-do 14662, Korea; jesseily92@gmail.com; 2 Department of Electrical Engineering, Pusan National University, Busan 46241, Korea; nik@pusan.ac.kr

**Keywords:** pixel-level ADC, current-input ADC, readout circuit, microbolometer, high SNR, wide dynamic range

## Abstract

A readout circuit incorporating a pixel-level analog-to-digital converter (ADC) is studied for two-dimensional medium wavelength infrared microbolometer arrays. The signal-to-noise ratio (SNR) and charge handling capacity of the unit cell circuit are improved by using the current input pixel-level ADC. The charge handling capacity of the integrator is appropriately extended to maximize the integration time regardless of the magnitude of the input current and low power supply voltage. The readout circuit was fabricated using a 0.35-μm 2-poly 4-metal CMOS process for a 640 × 512 array with a pixel size of 40 μm × 40 μm. The peak SNR and dynamic range are 77.1 and 80.1 dB, respectively, with a power consumption of 0.62 μW per pixel.

## 1. Introduction

Infrared cameras, which detect objects by infrared rays in a situation where they cannot be observed by human eyes, are widely used in military, medical, and commercial fields. In recent years, there has been an increase in the demand for compact and power efficient portable infrared cameras, especially in the private sector for medical devices and security cameras. Therefore, significant research has been conducted on microbolometers, which are uncooled-infrared detectors that do not require a cryogenic cooling system [1,2,3,4].

While designing a readout integrated circuit (ROIC) for infrared focal plane array (IRFPA), noise performance is of crucial concern. The dominant noise sources in a microbolometer are the Johnson noise and 1/f noise, which can be decreased by increasing the integration time [5]. Therefore, it is necessary to use a pixel-level readout architecture with a large integration capacitor to obtain a long integration time. However, a microbolometer has a high bias current compared to its signal current. Hence, it is hard to locate a large integration capacitor in the pixel because of area limitation. To effectively overcome this problem, a bias-current skimming technique can be used [6]. In addition to noise performance, the dynamic range (DR), which is defined as the ratio of the maximum allowable signal to the minimum detectable signal, is also an important factor. In many applications, either the frame rate is very slow or the target temperature varies over a wide range. Hence, it is necessary to consider both the noise performance and the charge handling capacity of the ROIC.

A monolithic analog-to-digital converter (ADC) is essential for compact and power-efficient portable infrared cameras. Information can be transported with a high signal-to-noise ratio (SNR) in a more power efficient manner in the digital domain using a monolithic ADC, and on-chip signal processing for system-on-chip (SoC) can be made available. Monolithic ADCs can be implemented at the chip level by employing a single high-speed ADC [7], at the column level by using multiple lower speed ADCs [8,9] or at the pixel level by using very low speed ADCs [10,11,12,13,14]. A pixel-level ADC has many advantages over chip and column level ADCs. These include low noise, low power dissipation, and the ability to continuously observe the pixel outputs for long time [14,15].

Although the pixel-level ADC has many advantages, conventional ADC architectures are not easily implemented in IRFPAs because a low-power, high-resolution ADC with small size is required. Since a single-slope ADC (SS-ADC) has a very simple structure, a pixel-level ADC can be easily implemented using this method [10]. However, since analog-to-digital (A/D) conversion is performed after integration, SNR and DR cannot be improved. In addition, a large number of comparisons are required to implement a high-resolution SS-ADC, which significantly increases power consumption.

Another method to implement a pixel-level ADC is based on the time-to-digital (TTD) ADC, also known as the time-to-first spike ADC [11]. While integrating the input current, it detects the time at which the integral signal reaches the reference voltage. Properly modifying the reference voltage can improve the DR characteristics. However, since the integration time varies depending on the input current, it is difficult to obtain a good SNR or a suitable digital resolution for a large input current.

Studies have been conducted that implement pixel-level ADC using pulse frequency modulation (PFM) method [12,13]. While integrating the input current, the integral signal is reset when the integral signal reaches the reference voltage, and A/D conversion is performed using the reset number. Since sufficient integration time and charge handling capacity can be obtained, SNR and DR characteristics can be improved. However, the PFM-based implementation requires many reset pulses to obtain a high-resolution ADC. Therefore, it has high power consumption and is disadvantageous for fast operation. To overcome these problems, the two-stage method can be a good solution [16]. However, since accuracy and linearity are affected by the reset interval, A/D conversion performance may be poor depending on the characteristics of the comparator. In addition, it is still difficult to reduce power consumption since the comparator is always on as the asynchronous method is used.

In the proposed readout circuit, current-input pixel-level ADC using a synchronous two-stage A/D conversion is efficiently performed during the integration, and the characteristics of power consumption, SNR, and DR are improved with high resolution.

## 2. Basic Concepts of the Proposed ADC

**Figure 1:**
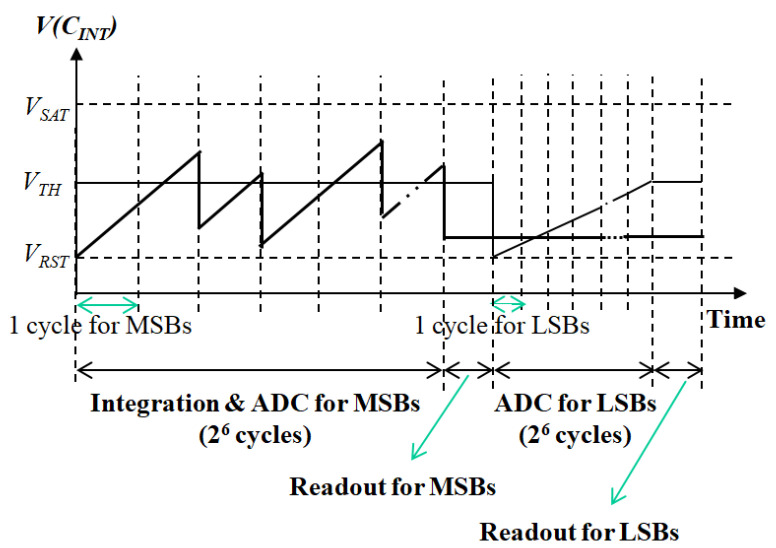
Basic concepts of the proposed pixel-level analog-to-digital converter (ADC).

The current input pixel-level ADC proposed in this paper uses a synchronous two-stage A/D conversion to reduce power consumption and improve SNR and DR characteristics. The basic concepts of the proposed ADC are explained in the timing diagram shown in Figure 1. First, the current input is integrated and converted into a voltage *V*(*C_INT_*), and it is compared with the reference voltage (*V_TH_*) at regular time intervals. *V_RST_* and *V_SAT_* denote the reset voltage and the maximum allowable voltage of *V*(*C_INT_*), respectively, and *V_TH_* is equal to 0.5(*V_RST_* + *V_SAT_*). If the value of *V*(*C_INT_*) is greater than the value of *V_TH_* at the comparison point, the constant voltage of 0.5(*V_SAT_* – *V_RST_*) is removed from *V(C_INT_*). This process is repeated periodically during the integration of the current input. The digital information *D_OUT_* on the current input *I_SIG_* is obtained by counting the number of removals and is expressed by

(1) $$D_{OUT}=\left[\frac{2T_{INT}I_{SIG}}{C_{INT}\left(V_{SAT}-V_{RST}\right)}\right]$$

where *T_INT_* is the integration time, *C_INT_* the integration capacitor, and the square bracket [] represents the function of digitization. This method is based on first-order incremental ADC [17]. Using this method, A/D conversion is efficiently performed during integration and a sufficient integration time and large charge-handling capacity is obtained. The maximum charge-handling capacity *Q_M_* is expressed by

(2) $$Q_{M}=2^{M-1}C_{INT}\left(V_{SAT}-V_{RST}\right)$$

where *M* is the resolution of the A/D conversion. Since a synchronized method is used where comparing *V*(*C_INT_*) and *V_TH_*, power consumption of the comparator is minimized.

In a typical application for medium wavelength infrared (MWIR) microbolometer, even if the current input is integrated after removing the bias current, the ADC of the readout circuit must have a resolution of 12-bit or more [12,14]. To implement a 12-bit resolution using only the first-order incremental ADC, 2^12^ comparison cycles are required, which is a great burden in terms of speed and power consumption. To solve these problems, a two-stage A/D conversion is used as shown in Figure 1. In the first period, A/D conversion for the upper six-bit is performed during the integration as described above. After the integration, the residual voltage of *V*(*C_INT_*) is held and the lower six-bit is determined by additional single-slope A/D conversion. In this case, since the number of the comparison cycles for implementing a 12-bit ADC is reduced to 2^7^ (2^6^ + 2^6^) times, a compact and low-power design is possible, which can be easily implemented as a pixel-level ADC. The circuits for the upper and lower six-bit A/D conversion are shared by dividing the time, so that the conversion result for the upper six-bit should be read out of the array before the lower six-bit conversion.

## 3. Circuit Implementation

**Figure 2:**
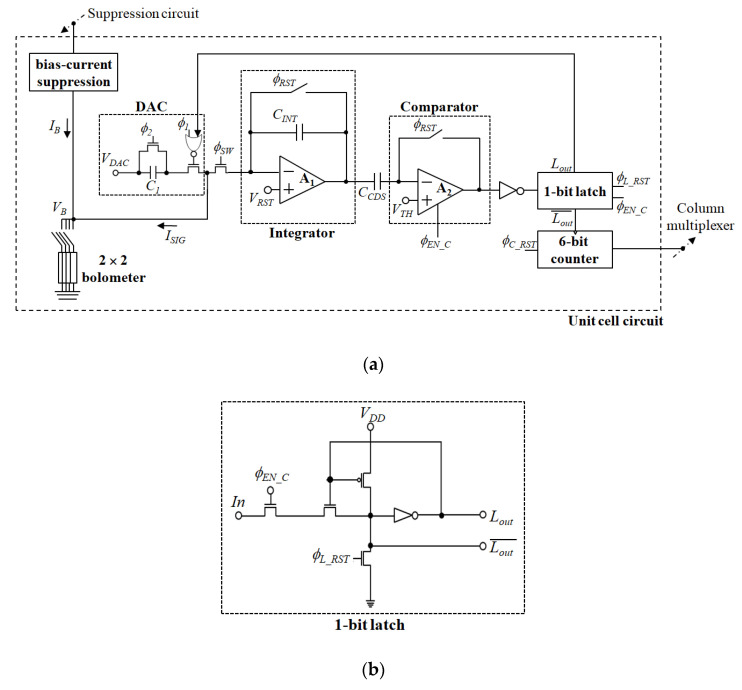
Schematic diagram of the proposed ADC: (**a**) overall unit cell circuit; (**b**) one-bit latch used in unit cell circuit.

**Figure 3:**
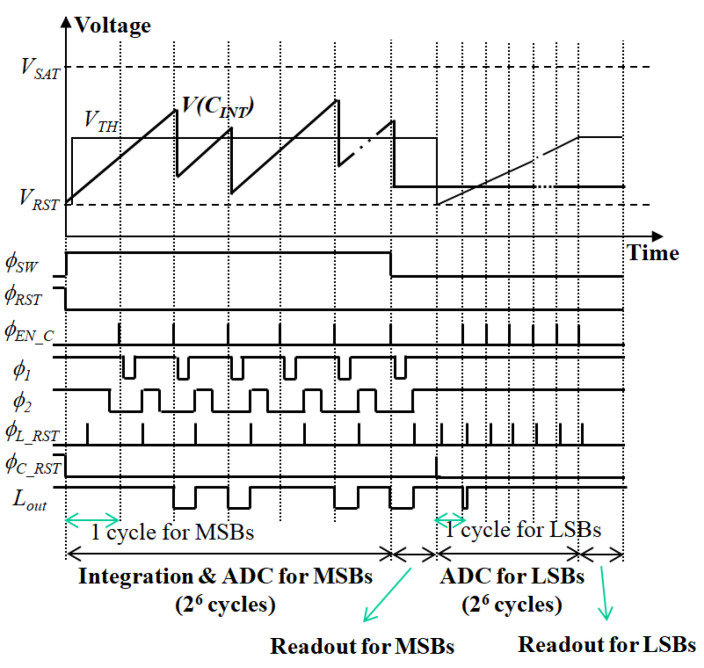
Timing diagram of the proposed ADC shown in Figure 2.

Figure 2 and Figure 3 show the schematic diagram and detailed timing diagram of the proposed pixel-level ADC, respectively. Since a microbolometer has a high bias current compared to its signal current, bias-current suppression is used [6]. Using the accurate bias-current suppression, the integration time can be increased to improve the SNR and the resolution required for A/D conversion can be reduced. Additionally, fixed-pattern noise (FPN), which is produced by the non-uniform IRFPA response, can be lowered to reduce the burden on non-uniformity correction [6,18]. The responsivity of the microbolometer is highly dependent on the bias voltage *V_B_*, hence, maintaining a stable bias voltage is crucial in microbolometer applications [19]. Therefore, a capacitive transimpedance amplifier (CTIA) is used for current integration even though it has a high-power consumption.

First, the current input *I_SIG_* is integrated with the integrator and the value of *V*(*C_INT_*) is compared with the value of *V_TH_* using the comparator. Here, *ϕ_SW_* and *ϕ_RST_* are control signals for determining the integration time and reset control signals for the integrator, respectively. The *C_CDS_* is the capacitor required for correlated double sampling (CDS) operation [20], reducing FPN and low-frequency noise and compensating for the offset voltage of the comparator and integrator. To completely eliminate all FPNs and non-uniformities in IRFPA and ROIC, an additional calibration process is required. The comparison result is transferred and stored in a one-bit latch according to the *ϕ_EN_C_* signal. The result of the comparison is a logic 0’, the output of the one-bit latch *L_out_* is a logic 0’, and the constant charge is removed from the integrator to the DAC according to the control signals of *ϕ*_1_ and *ϕ*_2_. The one-bit latch is then reset according to the *ϕ_L_RST_* signal for the next comparison cycle. The inversion signal of *L_out_* is used as a clock signal of a six-bit counter, through which the digital value of the upper six bits for the current input is determined. *ϕ_C_RST_* is used for the reset control signal of the six-bit counter.

After completing the integration and A/D conversion of the upper six bits, the *V*(*C_INT_*) value is held by using *ϕ_SW_*, and the digital values stored in the counter are sequentially transferred to the column multiplexer outside the array. A/D conversion of the lower six bits is implemented as a single-slope A/D conversion method for the residual *V*(*C_INT_*). First, the six-bit counter is reset using the *ϕ_C_RST_*, and the *V_TH_* value changes from *V_RST_* to 0.5(*V_RST_* + *V_SAT_*) in the form of a ramp signal. When the value of *V_TH_* is less than the value of *V*(*C_INT_*), the input of one-bit latch is logic 1’, and the *L_out_* changes periodically by *ϕ_EN_C_* and *ϕ_L_RST_*. Therefore, the digital value of the six-bit counter increases until the *V_TH_* exceeds *V*(*C_INT_*), and the final digital data stored in the counter is the lower six-bit digital data for the current input. Finally, the six-bit digital data is sequentially transferred once again to the column multiplexer outside the array.

**Figure 4:**
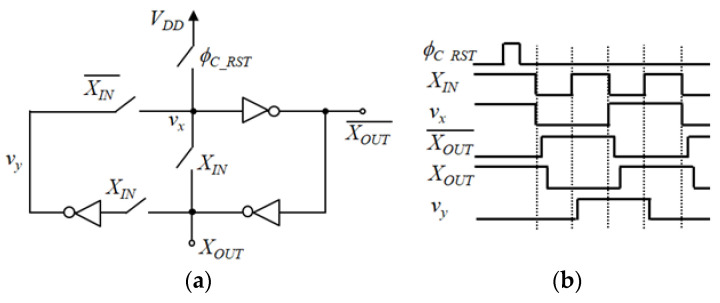
One-bit counter used in the unit cell circuit of Figure 2: (**a**) schematic diagram; (**b**) timing diagram.

Figure 4 shows a one-bit counter used in the unit cell circuit in Figure 2. It is a simple structure using three logic inverters, and it can be easily applied to the pixel-level ADC. *X_IN_* and *X_OUT_* are clock and output, respectively, and *X_OUT_* turns into the logic value of *v_y_* when *X_IN_* changes from logic 1’ to 0’. Due to the delay time between *X_IN_* and *X_OUT_* signals, *v_y_* is not affected by changes in *X_OUT_* signal at this moment. Subsequently, when *X_IN_* changes from logic 0’ to 1’, *v_y_* changes to the inversion of *X_OUT_*. The final six-bit counter is configured as an asynchronous ripple counter using six one-bit counters in Figure 4.

**Figure 5:**
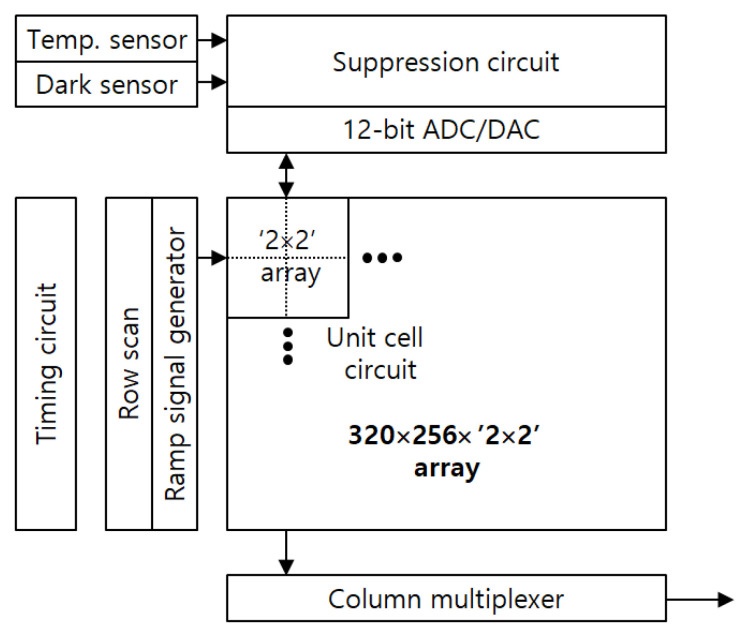
Block diagram of the overall arrangement of the proposed readout integrated circuit (ROIC).

Figure 5 shows the overall arrangement of the proposed ROIC. The unit circuit in Figure 2 has a simple configuration. However, it is difficult to implement the proposed unit circuit in one pixel of 40 µm × 40 µm. Thus, as shown in Figure 5, four adjacent (2 × 2 array) bolometers share one unit circuit in Figure 2 in a time-dividing manner. The size of the bolometer array is 640 × 512, and the unit circuit has an array of 320 × 256. The temperature sensor detects the substrate temperature, and the dark sensor obtains the reference value of the bias current. The suppression circuit and 12-bit ADC/DAC blocks are used to control the bias current of the bolometer array [6]. Timing circuit, row scan, and ramp signal generator are required to control the unit circuit, and column multiplexer is required to transfer digital data outside.

## 4. Experimental Results

**Figure 6:**
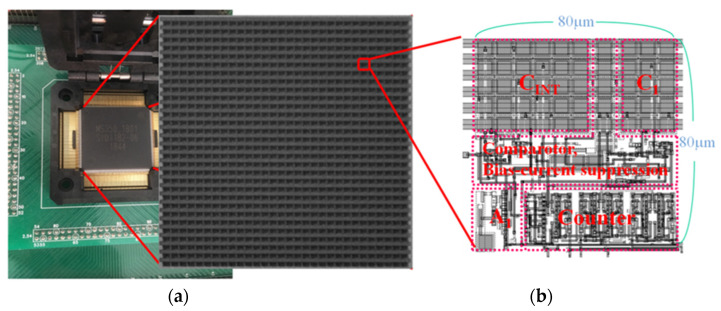
Measurement system: (**a**) operation board and fabricated chip; (**b**) mask layout of the proposed unit cell circuit for 2 × 2 bolometer array.

The readout circuit was designed using a 0.35 µm 2-poly 4-metal CMOS process for a 640 × 512 MWIR a-Si microbolometer array with a pixel size of 40 µm × 40 µm. The design parameters of the microbolometer and readout circuit are summarized in Table 1. The fabricated unit cell circuit is 80 × 80 µm^2^ in size because the proposed unit circuit is shared by 2 × 2 pixels. Uncooled microbolometers are difficult to operate at speeds above 60Hz. Therefore, the frame rate of IRFPA is set to 60 Hz and the sampling rate of the proposed pixel-level ADC is 240 Hz. It was tested at room temperature using the operation board shown in Figure 6.

**Figure 7:**
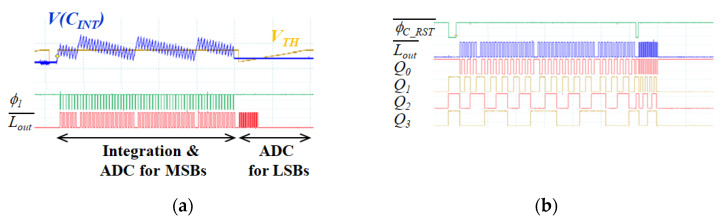
Measured operating waveforms of the proposed pixel-level ADC: (**a**) current integration and A/D conversion; (**b**) counter output.

Figure 7 shows the operating waveforms of the proposed pixel-level ADC. The current input is integrated and converted to MSBs in the first period. Subsequently, the residual *V*(*C_INT_*) is held and converted to LSBs. MSBs and LSBs are estimated by counting the number of $\bar{L_{OUT}}$. Figure 7 b shows the operating waveforms of the counter output (*Q*_3_*Q*_2_*Q*_1_*Q*_0_). Only four bits are displayed among six-bit outputs, and *Q*_0_ represents the least significant bit. As shown in Figure 7, the range of the available current input that does not saturate during integration time is drastically improved by the current input pixel-level ADC.

**Figure 8:**
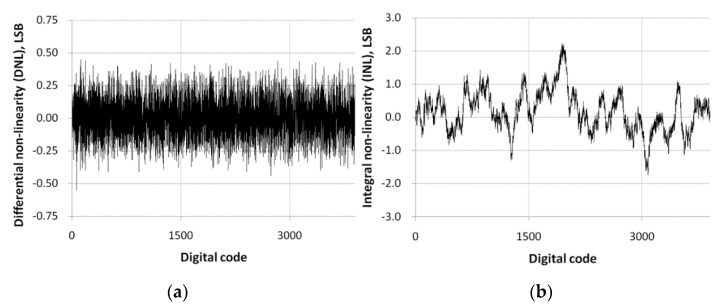
Measurement results of the linearity of the proposed pixel-level ADC: (**a**) differential non-linearity (DNL); (**b**) integral non-linearity (INL).

Figure 8a shows the measurement results of differential non-linearity (DNL). Figure 8b shows the integral non-linearity (INL) estimated by Figure 8a. The DNL is within about ±0.4 LSB, and the INL is within about ± 2.0 LSB. These results confirm the monotonicity of the proposed pixel-level ADC.

**Figure 9:**
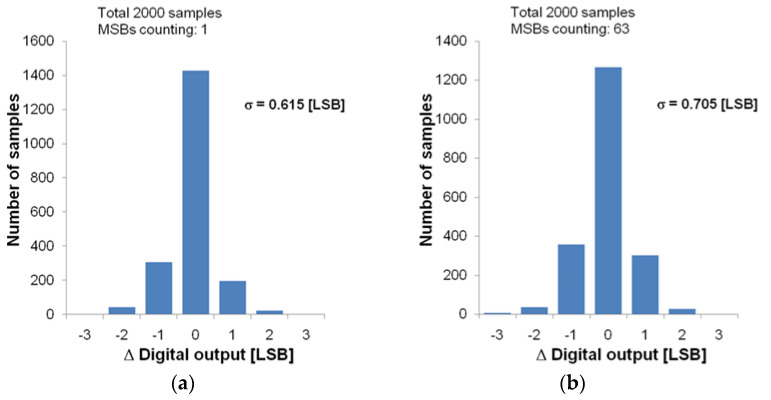
Histograms of the digital output of the proposed ADC with a fixed input: (**a**) where the number of MSBs counting is 1; (**b**) where the number of MSBs counting is 63.

**Figure 10:**
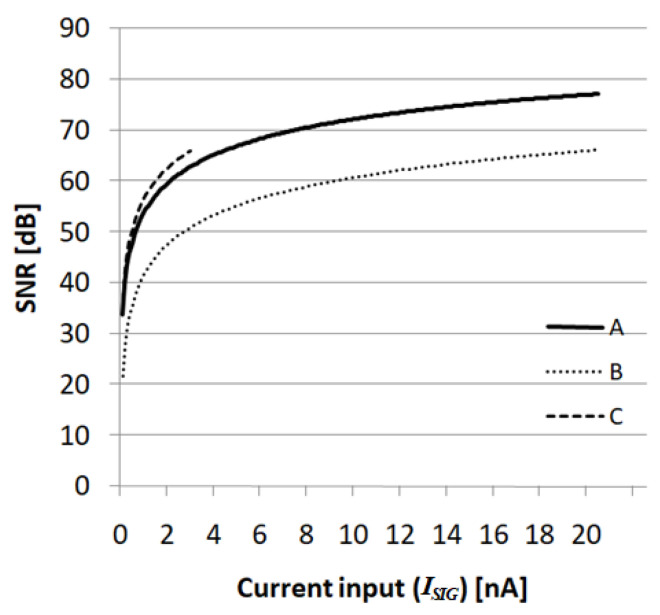
SNR characteristics for the three cases: (**A**) the proposed pixel-level ADC; (**B**) 12-bit SS-ADC with reduced integration time; and (**C**) 12-bit SS-ADC with reduced input range.

SNR and DR characteristics are important figures of merit for IRFPA applications. To estimate the SNR and DR characteristics, the readout circuit noise, i.e., the ADC noise, should be measured. First, the digital output of the ADC for a zero current input was sampled 2000 times, then the readout noise for a zero current input was estimated as the standard deviation *σ* of the sampled output. Through this process, it was confirmed that the readout noise for a zero-current input *σ*_0_ is close to 0 LSB. However, this method alone cannot evaluate the effect of kTC noise of the DAC on the readout noise. In order to evaluate the readout noise caused by the DAC, there must be a current input. In this case, the readout noise itself can be evaluated by eliminating the noise caused by the current input. The ADC output was sampled several times for two different current inputs, and the histograms were obtained as shown in Figure 9. Figure 9a,b are histograms obtained using a total of 2000 samples, where the number of MSBs counting is 1 and 63, respectively. Using the data in Figure 9, the noise of the current input system *σ_I_* can be removed, and the noise equivalent to one MSB counting *σ_M_* can be predicted as shown in following equation:

(3) $$\sigma_{a}^{2}=\sigma_{0}^{2}+\sigma_{I}^{2}+\sigma_{M}^{2}$$

(4) $$\sigma_{b}^{2}=\sigma_{0}^{2}+\sigma_{I}^{2}+63\sigma_{M}^{2}$$

(5) $$\sigma_{M}=\sqrt{\left(\sigma_{b}^{2}-\sigma_{a}^{2}\right)/62\;\;}=\;0.0438\;[\mathrm{LSB}]$$

where *σ_a_* and *σ_b_* are the *σ* of Figure 9a,b, respectively. After predicting the *σ_M_* value from the histograms of Figure 9, the readout noise according to the current input can be evaluated and applied to the final SNR characteristic of Figure 10.

Figure 10 shows the SNR characteristics according to the current input, and the total noise is estimated from the bolometer noise, quantization noise, and the readout noise. The legend A of Figure 10 indicates the graph of the proposed pixel-level ADC with a *T_INT_* of 3.1 ms and a maximum *I_SIG_* of 20.4 nA. Legends B and C indicate the case of applying the conventional 12-bit SS-ADC after the integration time. In the case of B and C, since the charge handling capacity is limited due to the pixel area limitation, it is necessary to reduce the integration time to 98 μs for a wide current input (B) or reduce the input range to 3.0 nA for a long integration time (C). Design parameters except for *T_INT_* and maximum *I_SIG_* are the same as in Table 1 for A, B, and C. Since the charge handling capacity of the proposed ADC is appropriately extended, the integration time is maximized for a wide input range. Therefore, the proposed ADC has good SNR characteristics for a wide input range as shown in Figure 10.

Table 2 shows the performance comparison between the proposed ADC and other methods. The design of the readout circuit for IRFPA is greatly dependent on the IR detector. Therefore, in the case of A, A′, B, and C, the main design parameters are the same for reliable comparison. A’ indicates the case of applying the proposed ADC without bias-current suppression. Since the maximum value of the bias current is about 60 nA, if the bias current is not suppressed, the integration time decreases and both charge handling capacity and SNR decrease. Without bias-current suppression, the effective signal range decreases and the burden required for non-uniformity correction is increased [12,14]. Therefore, the resolution of the ADC must be increased in the actual design. Although it is difficult to make a clear comparison, the results presented in other papers are compared Table 2. Reference [10] uses the SS-ADC for pixel-level ADC and shows very low charge-handling capacity like B and C. Since the readout circuit of the reference [10] is for a cooled type MWIR detector, the bias current is small and the readout noise is very low. In addition, since the SNR value of A includes the noise of the detector, it is difficult to clearly compare the SNR values of A and [10]. The reference [11] uses the TTD method for pixel-level ADC, and the proposed ADC shows superior SNR characteristics. Since the readout circuit of the reference [11] is for a short wavelength infrared (SWIR) detector, an additional method is used to increase the charge handling capacity to an extreme. It can be seen from the Table 2 that the proposed ADC shows excellent and balanced characteristics in both charge handling capacity and SNR.

**Figure 11:**
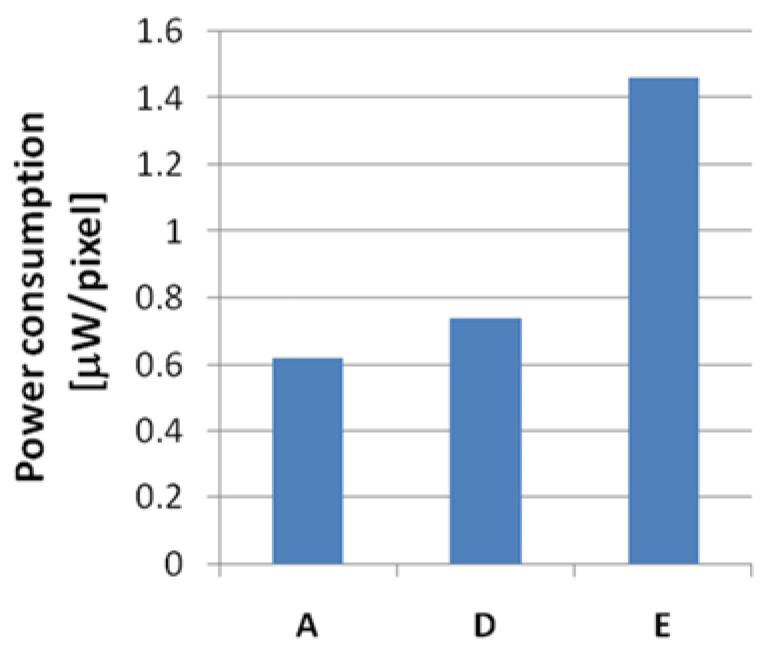
Power consumption for the three cases: (**A**) the proposed pixel-level ADC; (**D**) pulse frequency modulation (PFM)-based two-stage analog-to-digital (A/D) conversion; (**E**) 1st-order incremental ADC.

Figure 11 is a comparison of the results of the power consumption for the three cases, simulated using the same MOS library. The three cases are designed based on the same microbolometer shown in Table 1, and the bias-current suppression method is applied to all three circuits. Legend A indicates the power consumption of the proposed ADC, and D indicates the case of applying a PFM-based method to the A/D conversion for the upper six bits. Legend E indicates a case where the entire A/D conversion is implemented with only first-order incremental ADC. Considering the characteristics of the comparator and the maximum integration time, only 10-bit conversion is applied instead of 12 bits in the case of legend E. Since the comparator is always on as the asynchronous method is used in the case of D, it is still difficult to reduce power consumption. The 1st-order incremental ADC requires many comparison cycles to obtain a high-resolution ADC. Therefore, it has high power consumption. The proposed ADC can expect the lowest power consumption among the three methods as shown in Figure 11.

## 5. Conclusions

The current-input pixel-level ADC with a high SNR and wide DR is proposed for the readout circuit of the MWIR microbolometer array. In the proposed readout circuit, A/D conversion is efficiently performed during the integration, and a sufficient integration time and large charge-handling capacity is obtained. Using a synchronous two-stage A/D conversion, the characteristics of the power consumption, SNR, and DR are improved with high resolution. The proposed pixel-level ADC has a very simple structure and low power consumption. This enables the final readout circuit to be suitable for applications in portable infrared cameras.

## Figures and Tables

**Table 1:** Design parameters of the microbolometer and readout circuit.

Parameter	Value
Resistance	10 MΩ
Temperature coefficient of resistance	2.5%
Thermal conductance	5 × 10^−8^ W/K
Fill factor	80%
Emissivity	80%
Frame rate	60 Hz
Average bias current (*I_B_*)	40 nA
Maximum signal current (*I_SIG_*)	20.4 nA
Integration time (*T_INT_*)	3.1 ms
Integration capacitor (*C_INT_*)	1 pF
Power consumption	<0.62 µW

**Table 2:** Performance comparison.

	Charge Handling Capacity [e-]	Peak SNR [dB]	Description
**A**	405 M	77.1	Proposed ADC
**A’**	101M	74.6	Proposed ADC without Bias-current suppression
**B**	12.5 M	66.2	SS-ADC with reduced *T_INT_*
**C**	59.6 M	66.0	SS-ADC with reduced *I_SIG_* range
**[10]**	10 M	89.6	SS-ADC
**[11]**	1.98 G	60.9	TTD
**[12]**	310 M	>60	PFM

## Data Availability

Not applicable.
